# Knowledge, accessibility, and utilization of insecticide treated nets among pregnant women in a selected hospital in South-Eastern Nigeria

**DOI:** 10.18332/ejm/130591

**Published:** 2020-12-18

**Authors:** Chiamaka J. Okafor, Ngozi P. Ogbonnaya

**Affiliations:** 1 Department of Nursing Sciences, University of Nigeria, Nsukka, Nigeria

**Keywords:** pregnancy, malaria, mosquito, insecticide treated nets

## Abstract

**INTRODUCTION:**

Prevention of malaria in pregnancy (MIP) with the use of insecticide treated nets (ITNs) is no doubt one of the major interventions aimed at reducing maternal and infant morbidity and mortality rates. This study aimed to determine the knowledge, accessibility and utilization of ITNs during pregnancy.

**METHODS:**

This is a quantitative survey conducted in 2014 among pregnant women attending ANC in Redeemer Hospital and Maternity, Abakpa-Nike, Enugu State, South-Eastern Nigeria. One hundred and forty participants were selected using convenience sampling and information elicited using a self-developed questionnaire. Data were analysed using SPSS (version 9) and results presented in frequency tables.

**RESULTS:**

Among the respondents, 90.7% knew of the effectiveness of ITNs in the prevention of MIP. Results also showed that most of the women (69.3%) own at least one ITN, and their major source was from the free house-to-house distribution by the government. Also, 62.9% revealed that the ITNs were readily available. Out of the 97 women that owned at least one ITN, the majority (69.1%) claimed to have started using ITNs even before pregnancy with 70% claiming to sleep under the ITNs always. Nevertheless, only 69.1% used an ITN correctly.

**CONCLUSIONS:**

There is high knowledge of ITNs and their effectiveness in prevention of MIP among pregnant women in Enugu South-Eastern Nigeria. However, there is a need for measures to increase awareness on their correct usage, and also to correct misconceptions and wrong beliefs associated with ITNs in order to enhance their utilization.

## INTRODUCTION

Malaria has been a world health burden and a major public health problem in tropical and subtropical regions of the world. It affects an estimated 350 to 500 million people annually and accounts for 1 to 3 million deaths per year worldwide^1-3^. In its periodic health report, the World Health Organization (WHO) stated that more than 90% of the world’s malaria cases (an estimated 0.8 million annually) are unreported^4^. In African countries with stable malaria transmission, infection during pregnancy is estimated to cause as many as 10000 maternal deaths and 200000 neonatal deaths per year. It is also said to be responsible for 8–14% of all low birthweight babies and 3–8% of all infant deaths, and over 30 million pregnancies threatened throughout Africa each year^5-7^. In 2007, there were an estimated 219 million cases of malaria in 87 countries, while the number of deaths stood at 0.435 million^8^.

Malaria infection during pregnancy is a significant public health problem with substantial risks for the pregnant woman, her foetus, and the newborn child. Pregnant women have attacks of malaria more often and more severely than non-pregnant women from the same area, and are more likely to die from malaria. This is because pregnancy reduces women’s ability to fight malaria, especially during a woman’s first pregnancy^9^. About 19–24 million women are at risk of malaria during pregnancy^10^. Malaria-associated maternal illness and low birthweight are mostly the result of *Plasmodium falciparum* infection and occurs predominantly in Africa^11^. Malaria in pregnancy also contributes to significant peri-natal morbidity and mortality. Malaria infection is known to cause higher rates of miscarriage, intrauterine demise, premature delivery, low birthweight neonates, and neonatal death^12^.

Prevention of malaria during pregnancy with the use of ITNs is no doubt one of the major interventions aimed at reducing maternal and infant morbidity and mortality rates and thus achieving the 4th, 5th and 6th Millennium Development Goals (MDGs). It has remained a cost effective and highly efficient tool in global and national malaria control policies^13,14^ and it is estimated to be twice as effective as the untreated nets5. The 2008 Nigeria Demographic and Health Survey (NDHS) results indicate that 17% of households in Nigeria own a mosquito net (treated or untreated), and 8% of households own more than one mosquito net and 16% of households own at least one net that has never been treated. The average number of ITNs per household was less than one, which could be attributed to a weak supply and distribution mechanism^5^.

In recent years, the distribution of ITNs has been inadequate, with only a few local government areas targeted in various states. This has made it impossible to attain saturation in any one area^5^. The approach since 2009 has been to start afresh a coordinated strategy to deliver 2 nets to every household across the country through a series of stand-alone campaigns to achieve universal coverage. In 2010, the world-bank booster-supported states (Kano, Jigawa, Bauchi, Gombe, Anambra, Akwaibom, and Rivers) conducted net campaigns, and health workers distributed free nets to households. The aim was to promote net-use in households, especially among pregnant women and children below 5 years of age^5^. According to the Federal Ministry of Health, 57.7 million nets were distributed between 2009– 2013 across Nigeria, representing 90.2% of the national overall coverage target^15^. This coverage represented a huge success in the collective efforts to scale up the intervention.

Again, anecdotal reports from both tertiary health facilities and primary healthcare centres in Enugu, South-Eastern Nigeria, show that the use of ITNs is somewhat limited. Our literature review and observations also show that documentations on the knowledge, accessibility, and use of ITNs by this vulnerable group are also limited. It is not known whether the poor usage observed is due to a lack of knowledge about ITNs and their importance, or because access to ITNs is poor, or because there are other constraining factors. It is also not known whether usage and opinions about access to ITNs have improved over time. These issues motivated this study among antenatal care attendees of Redeemer Maternity, Abakpa Nike, Enugu, South-Eastern Nigeria to determine the knowledge, accessibility and utilisation of insecticide treated nets during pregnancy.

## METHODS

The current quantitative study was undertaken at Redeemer Hospital and Maternity, Abakpa Nike, Enugu State, South-Eastern Nigeria. The study population consisted of all the pregnant women who attend the antenatal care clinic within 6 weeks of the study. One hundred and forty pregnant women were recruited and included in the study. The inclusion criteria included: 1) must be a pregnant woman; 2) must be attending ANC at Redeemer Hospital and Maternity, Abakpa Nike; and 3) must be willing to participate in the study. On the other hand, the exclusion criterion entailed those who refused to sign the consent form.

The proposal for this study was reviewed and approved by the hospital ethics board. Participants were informed about the purpose and details of the study and written consent was obtained from them prior to the commencement of the study. A self-developed questionnaire was used for the study. The questionnaire was constructed based on the research objective. The questionnaire had two sections. Section A comprised the sociodemographic data, while Section B comprised questions or items set to generate data for the research, based on the research questions. Most of the questions were designed in close ended format, which gives the respondent the opportunity to choose from the options provided, while others were in open ended format where the respondents were expected to write their own opinions.

The reliability of the instrument was tested by carrying out a pilot survey in which 25 copies of the questionnaire were administered to 25 pregnant women at Annunciation Specialist Hospital, Emene, Enugu East LGA. Data generated were tested for internal consistency (the degree to which all of the items measure a common characteristic). A reliability coefficient of 0.89 was obtained using Cronbach’s alpha. Thus, the instrument was considered reliable. Data were analysed using SPSS (version 9) and are presented in frequency tables.

## RESULTS

The demographic profile of participants is presented in Table 1. All the respondents had knowledge of ITNs and 90.7% knew of their effectiveness in the prevention of malaria in pregnancy. The majority (45%) obtained the information by attending antenatal clinics; only a few (4.3%) were informed by their family (Table 2). Results also show that most of the women (69.3%) own at least one ITN, and their major source was from the free house-to-house distribution by the government. Also, 62.9% revealed that the ITNs were readily available (Table 3).

**Table 1:** Sociodemographic characteristics of respondents (N=140)

*Characteristics*	*Categories*	*n*	*%*
**Age** (years)	<20	9	6.4
	20–29	94	67.1
	30–39	37	26.5
	≥40	-	-
**Highest educational level**	No formal education	-	-
	Primary	18	12.9
	Secondary	74	52.8
	Tertiary	48	34.3
**Parity**	1	47	33.6
	2	53	37.8
	3	20	14.3
	4	12	8.6
	≥5	8	5.7
**Religion**	Christian	140	100
	Islam	-	-
	Traditional worshipper	-	-
**Stage of pregnancy**	1st trimester	26	18.6
	2nd trimester	75	53.6
	3rd trimester	39	27.8
**Occupation**	House wife	59	42.1
	Petty trader	42	30.0
	Artisan	12	8.6
	Civil servant	19	3.6
	Student	8	5.7
**Number of malaria episode occurrence(s) during current pregnancy**	0	46	32.9
	1	64	45.7
	2	27	19.3
	3	3	2.1
	≥4	-	-

**Table 2:** Knowledge of pregnant women regarding prevention of malaria in pregnancy using ITNs, Nigeria (N=140)

*Items*	*n*	*%*
**Do you know about ITNs**		
Yes	140	100
No	-	-
**How did you get to know about ITNs**		
Antenatal care centres	63	45.0
Health education campaigns	21	15.0
Mass media (radio, TV, newspapers, etc.)	43	30.7
Friends and colleagues	7	5.0
Family	6	4.3
Neighbours	-	-
**Why do you think ITNs are important during pregnancy**		
They reduce the contact between the pregnant women and mosquitoes	127	90.7
They provide a house for the mosquitoes to stay in	8	5.7
They drive away mosquitoes from the room	5	3.6
It is an instruction from the antenatal clinic that must be followed	-	-

**Table 3:** Accessibility to ITNs by pregnant women, Nigeria (N=97)*

*Items*	*n*	*%*
**Do you own at least one ITN**		
Yes	97	69.3
No	43	30.7
**What is the source of your ITN**		
Antenatal care centres	15	15.5
Market	15	15.5
Friends/relatives	22	22.6
Free house-to-house distribution	30	30.9
Pharmacy	15	15.5
**Were ITNs readily available for you**		
Yes	61	62.9
No	36	37.1

* Responses from only participants who possessed at least one net.

### Utilisation of ITNs by pregnant women

Out of the 97 women that owned at least one ITN, the majority (69.1%) claimed to have started using ITNs even before pregnancy with 70% claiming to sleep under the ITNs always. Regarding the correct use of an ITN, 69.1% stated that they used it correctly, hanging it over the bed and tucking it under the mattress to prevent contact with the body while sleeping under it. Others stated that they hung it over their doors and windows to prevent mosquitoes from entering their rooms (17.5%), while only a minority still allowed contact with the net while sleeping under it (13.4%). Multiple issues were found that prevent correct usage of ITNs. For example, 41 (95.3%) said they had heard that ITNs have a negative effect on the baby in the womb. Furthermore, 39 (90.7%) said they did not know how to hang it and in any case had no facility to hang it in their room. The heat was also an issue and that the ITNs cause excessive heat and discomfort, with 32 (74.4%) saying they cannot use them when the weather is hot. Furthermore, free distribution of ITNs was found to be a well-appreciated intervention that can increase its utilisation, as reported by 97.7 % of the participants (Table 4).

**Table 4:** Utilization of ITNs by pregnant women, Nigeria (N=97)

*Items*	*n*	*%*
**When did you start using ITNs**		
Before pregnancy	67	69.1
During 1st trimester	16	16.5
During 2nd trimester	7	7.2
During 3rd trimester	7	7.2
**Frequency of use**		
Always	68	70.0
Often	9	9.3
Sometimes	20	20.7
**How did you place your ITN**		
Hanging it over the windows and doors to prevent mosquitoes entering the room	17	17.5
Hanging it at one corner of the room	-	-
Hanging it over the bed, but the net has contact with the skin while sleeping under it	13	13.4
Hanging it over the bed and sleeping under it after tucking it under the mattress, preventing contact with the body	67	69.1
Constraining factors to correct usage of an ITN*		
It is too costly, I cannot afford it	6	13.9
It causes excessive heat and discomfort	32	74.4
I don’t know how to hang it	39	90.7
There is no facility to hang it in my room	39	90.7
I can’t use it when the weather is hot	19	44.2
The routine of raising it every morning after use is discouraging	13	30.2
I heard that ITNs have a negative effect on the baby in the womb	41	95.3
I did not get it when it was freely distributed	38	88.4
It traps mosquitoes inside when tucked in	4	9.3
Enhancing factors to correct the use of ITNs*		
Adequate information on how to hang ITNs should be provided	39	90.7
They should be made readily available	39	90.7
They should be given free-of-charge	42	97.7
The price should be reduced	26	60.5

* Responses on constraining and enhancing factors are mutually exclusive.

## DISCUSSION

### Knowledge of ITNs for prevention of malaria in pregnancy

The findings revealed that the majority of the respondents knew about ITNS and their importance in the prevention of malaria in pregnancy (MIP). This response is not surprising due to the high ongoing public awareness campaign on ITNs. Awareness is also created by the community health extension workers on the importance of ITNs. Even in most primary healthcare centres, the government has provided these nets to be distributed free-of-charge to pregnant women. Their source of information revealed that many became familiar with the ITNs through antenatal care centres. Only a few heard about them from friends and family members. In order to achieve greater awareness, there is a need to expand public knowledge on the importance of ITNs through targeted and multimedia approaches. This is in contrast to the study of Olayemi et al.^16^ which revealed that the majority obtained information about ITNs from friends and newspapers, while none had any health education in the clinic.

Responses to further questions regarding the importance of ITNs during pregnancy revealed that the majority knew that ITNs reduce the contact of the pregnant woman with mosquitoes. These responses indicate that knowledge about ITNs is very high. This is also not surprising because, from the demographic profile, the majority attained a secondary or tertiary education level. The high literacy level, no doubt, played a significant role in their knowledge. Being educated, they are able to comprehend information provided by the newspapers and other mass media. This emphasises the need for measures to improve the educational level/status of women, especially when they are still girls. This result is different to that of Musa et al.^17^ which showed that only one-third of the respondents were aware of ITNs in malaria prevention, indicating that the knowledge was low.

### Accessibility of ITNs to pregnant women

Findings revealed that the majority of the respondents own ITNs. This is in contrast to the findings of Ugwu et al.^18^ on the utilisation of ITNs among pregnant women in Enugu, South-Eastern Nigeria, which showed that only a few of the respondents (43.1%) owned ITNs. This shows that over the years, there has been a remarkable improvement in the accessibility of ITNs to pregnant women.

Findings also revealed that among the respondents that owned ITNs, the majority got their own during the free house-to-house distribution by the government. Others obtained them from public healthcare centres, pharmacy shops, markets, and friends. Further analysis also reveals that the ITNs were also readily available so that people could purchase them. There have been increased efforts by the government to make ITNs readily available and accessible to pregnant women; the primary healthcare facilities especially are provided with an adequate supply of ITNs that are freely distributed. This makes it easy for this vulnerable group, irrespective of social class, to own at least one ITN. This is however in contrast to the finding of Olayemi et al.^16^ which showed that 62.2% indicated that ITNs were not readily accessible. There is a need for the government to ensure the safe provision and free distribution of the ITNs. Because of the large size of the ITN market there has also been private production and sale of these nets at affordable prices, which has supplemented the government’s efforts to supply the families that missed out on the free nets from the government.

### The utilisation of ITNs by pregnant women

The findings revealed that out of the 97 women that own at least one ITN, the majority started using them even prior to current pregnancy, and 70% claimed to use an ITN always. Further responses also revealed that most of the women used the ITNs correctly by hanging them over their beds and preventing contact with the body while sleeping underneath. This finding goes a long way to reveal that the efforts made towards creating awareness regarding ITNs have been successful so far. Most people are now sensitised and aware of their own responsibilities to properly use the ITNs in order to benefit from them.

The major constraint to the use of ITNs was a belief that they may have a negative effect on the woman and the unborn baby, despite the high knowledge of ITNs effectiveness for prevention of MIP. Though there is high knowledge/awareness about ITNs, some people have not adequate knowledge to remove misconceptions surrounding ITNs to enable them to accept and use ITNs, especially those whose sources of information are friends and colleagues, instead of the health centres where reliable information is available. This assertion is supported by the low percentage of respondents that indicated that they had been adequately made aware. This is contrast to the findings of Olayemi et al.^16^ where only a minority believed that the treated net may have negative and adverse effects on them and their baby. Other reasons were: difficulty in hanging it properly over the bed, and excessive heat and discomfort caused by sleeping under ITNs. Better instructions on how to properly hang ITNs should be given to ensure correct usage. It is also suggested that the ITNs should be distributed free-of-charge or subsidised.

### Limitations

The limitation of the current study is the small sample size. The study included 140 participants selected at convenience and this might limit its generalizability.

## CONCLUSIONS

All pregnant women were ready to use ITNs if adequate information was provided and the nets made readily available at an affordable price or provided free-of-charge; thus, the government is encouraged to continue the provision of ITNs to attain a broader coverage. Health workers at all levels, especially primary level, are also encouraged to increase awareness of the correct usage of ITNs to ensure their full benefit.
